# The doctor, the patient, and the computer

**DOI:** 10.46989/001c.121434

**Published:** 2024-07-23

**Authors:** Finn Bo Petersen, Mohamad Mohty, Didier Blaise

**Affiliations:** 1 International Academy for Clinical Hematology; 2 Institut Paoli-Calmettes

**Keywords:** art of hematology, Doctor–Patient Relationship

As senior hematologists and cell therapists with more than 100 years of experience in this area between us, we receive many letters and e-mails from patients. Some of these graciously thank us and the staff for excellent care, and some are complaints about “something that went wrong” in their care experience. A new, but increasingly recurrent theme of the complaints we have received in the last few years is about *“the doctor kept looking into the computer screen on their desk during the whole consultation and wasn’t looking at or listening to me”*.

This development has become an increasing concern to us, as we always try to communicate our belief to our students, residents and fellows, that a clinician must continuously focus on the patient, and intently listen to his/her whole story to understand why he/she is in front of us. We have taught that this is required for understanding how to apply and/or adjust our medical science and knowledge to heal, treat and, hopefully, cure the patient’s ailment. From this comes the ability to genuinely empathize with any patient. The latter is crucial to the oncologist’s ability to to communicate his/her skills and expertise to enable the patient to make a truly informed choice. Subsequently, we, physicians, must work as hard as we can to make the patient’s chosen option successful, even if that choice would not have been our first. While some may quibble with whether this meets a definition of practicing the *Art of Oncology*, few will likely disagree with the concept that a skillset as detailed in **Table 1** could be very useful in the general clinical practice.

Table 1. **Helpful talents to have when practicing the art of oncology**

1. Knowing the state of the art of general medicine, and medical science.
2. Having resilient curiosity and persistence in pursuing the best explanations for an individual patient’s unique and often complex challenges.
3. Understanding the patient’s ‘story’ and what that means for applying or modifying the science.
4. Having the ability to understand and genuinely empathize with any patient
5. Having excellent communication skills and the ability to make a patient make a truly informed choice.
6. Of options presented to the patient, always respect his/her choice, even if it is not the practitioner’s first choice, and work hard to make that choice successful for the patient as if it had been the practitioner’s first choice.

It is not lost on us, that the skills listed in Table 1, do not require excellence in computer data entry, programming, or management. Thus, we wonder, how it has come to be that the focus on the *practice* of oncology has become computer-based at an increasing pace, begging the question as to what impact this computerization has on patients, physicians, nurses, and other ancillary staff devoted to the treatment of cancer patients.

It is also of note, that several issues involved in incorporation of computerization into clinical medicine are shared between the ‘most developed’ and ‘less developed’ countries, making the issues universal.^1–3^ Indeed, the story of the impact of medical practice computerization on clinical care is quite similar in every country adopting some brand or version of an ‘electronic medical record’ (EMR) system, including the dynamics pushed forward by EMR software companies, which have successfully managed to convince multiple state and local governments, hospitals and clinics world-wide of the virtues of implementing their EMR system.^4,5^ In the USA, this resulted in the passage of the so-called HITECH Act in 2009,^6^ mandating healthcare providers to convert all medical charts to a digital format, a process which was completed 5 years later, when almost 100% of hospital facilities had implemented an EMR system, including most private practices and clinics.^7^ The European Union (EU) has passed similar decrees related to EMR systems used in EU countries, regarding the need to be able to electronically communicate patient information between the member countries.^8^ The goal was to allow all EU citizens and their providers to have electronic access to medical healthcare in any member country where they have received medical care.^9^

The selling points pushed by these EMR software manufacturers was that ‘interoperability’ would make it easy for clinicians everywhere to access health information on a patient, regardless of where and by whom that care was provided, claiming that this would greatly improve the quality of care and communication.^10^ However, the reality in 2024 is that little-to-no interoperability exists between the different EMR systems (though it does exist within some of the individual systems).^11^ A promise was also made that the EMR systems would eliminate medical errors. However, whereas there has been a decrease in some types of medical errors after EMR implementation, there has also been an increase in a new category of EMR-related errors. Whether these types of errors negate each other, or whether there is more of one than the other type, has not yet been unequivocally teased out.^12^ The EMR system also promised to improve patient engagement in the decision making about their own care, by allowing more face-to-face (FTF) time with their physician. However, the reality today is that clinicians spend more time entering data than they do conversing with and examining patients.^13^ It has been well documented that FTF time with patients has been lost in order for physicians to satisfy their computer requirements after implementations of EMR systems.^13,14^ Also, better and more accurate communication was promised, but has not materialised, because erroneous and out-of-place information from aggregate data entry errors (mostly due to hasty ‘copying-and-pasting’) is prevalent,^15,16^ and often difficult or even impossible to correct.^17^ Lastly, a promise was also made of enhanced privacy and security of patient data. However, as of Jan 31, 2023, in the USA alone, there have been more than 5,000 data breaches, each involving more than 500 patient records since 2009, totalling more than 500 million records.^18^ The full damage to patients from these breaches is still unfolding, and remains unclear.

After the EMR systems were implemented in the larger hospitals and organizations, anthropologic observational studies were carried out on how this affected the physician/nurse-patient relationship. In one such study,^19^ it was concluded that the EMR systems are structured not to prioritize patients, but to prioritize corporate stakeholders, being often incongruent with good clinical care. The authors argued that “*the EMR enforces the centrality of market principles in clinical medicine, redefining the clinician’s role to be less of a medical expert and more of an administrative bureaucrat, and transforming the patient into a digital entity with standardized conditions, treatments, and goals, without a personal narrative”*. Those of us who have been through the transition from paper charts to EMRs certainly recognize at least some truth in this contention.

The medical community has known for a long time what sort of care patients desire. The Picker Institute is a leading patient protection and healthcare charity in the United Kingdom (UK), and highly considered both in Europe and the USA.^20^ This institute has defined 8 principles of person-centred care (none of which should come as any surprise to any practising physician), which constitute the approach widely recognised as the preferred model of care:

- Fast access to reliable healthcare advice
- Effective treatment delivered by trusted professionals
- Continuity of care and smooth transitions
- Involvement and support for family and caregivers
- Clear information, communication, and support for self-care
- Involvement in decisions and respect for preferences
- Emotional support, empathy, and respect
- Attention to physical and environmental needs

Again, it is not lost on us that none of the above principles requires the implementation and use of medical computer technology. For the patients, the promise of EMR implementation overlap with the promises made to the physicians, such as “*all providers will have instant access to your medical record no matter where you show up, which is beneficial in emergencies”*.^21^ However, if a patient receives care in a facility with a different EMR system, getting this information is often time consuming, frequently taking days to weeks, can be costly to the patient, and is often provided as reams of disorganized computer printouts.^22^ Also, the patients are promised that there would be a decrease in errors in their electronic data, but several studies have shown this being unlikely. For example, Sigall *et al* showed that, of the thousands of patients who received survey invitations to read their EMR online, the 22% who responded found factual mistakes; 42% and 10% of these reported that the mistake was serious or very serious, respectively.^23^ Furthermore, as discussed above, an increasing number of patients have noted that their physicians have become increasingly distracted by the computer, which is often physically placed between the physician and patient, with the physician appearing to spend more time intently reading and entering patient data, than actually looking at, and conversing with the patient.^13,14^

In the USA as well as in several other countries, a government mandate has required for all patients to have real-time access to all test results and examination reports through an electronic ‘portal’. The Office of the National Coordinator for Health Information Technology (ONC) of the USA Department of Health and Human Services, made it clear in 2021 that providers can no longer delay, block or change the release of electronic health information to patients, including their laboratory, pathology, and imaging results, as well as affiliated and clinical notes.^24^ In a Viewpoint article published in 2023,^25^ Bellizzi discussed the implications of delivering “bad news” to patients through a technical computer-generated report, with no human health care provider involved and readily available to explain the implications of this information, often for days. He, himself, had a diagnosis of cancer, and had a follow-up CT scan on a Thursday, the report from which the following text was posted in his portal on late Friday afternoon: “*there is a moderate-sized focus of moderate intensity uptake in the lower neck, midline and slightly to the left of midline. Focal uptake in the lower neck may represent recurrent disease”*. He knew he had probably relapsed; however, it was written in a way that required the knowledge of a physician to understand the meaning. Dr Bellizzi discussed the possible panic and anxiety that could be experienced by a medically less sophisticated patient reading such a report on a Friday afternoon.

Furthermore, in addition to accessing generated medical information from the patients’ tests and visits through these portals, an increasing number of tasks are now required to be performed through the portal, such as scheduling, prescription refills, reading after visit summaries, etc. adding complexity which, at a minimum, requires some degree of computer literacy, without addressing whether our patients’ computer literacy is keeping pace. In May 2018, the USA Department of Education published a description of US adults who are not digitally literate.^26^ That report used data from the 2012 Program for the International Assessment of Adult Competencies (PIAAC), in which the computer illiteracy in adults in the USA aged 16-65 years was compared to that of other developed countries. The lowest rate of computer illiteracy (11-14%) was in the Nordic countries (Netherlands, Sweden, Norway, and Denmark), followed by the UK, USA, Belgium, Canada, and Germany (15-18%), and went up to 37% for Japan and 50% for Poland. The international average was 23%. While this is likely to have improved since 2012, it stands to reason that a substantial proportion of patients have some degree of computer illiteracy. The study showed an inverse relationship between computer illiteracy and patient age, resulting in the percentage of illiteracy increasing from 8% in the 16-24-year-olds to 28% in the 55-65-year-olds, which is problematic, because most of patients with cancer are over the age of 55 years. While the computer literacy of ‘aging’ younger patients is likely to have increased, we must take into consideration that the inverse is happening to patients beyond age 65 years of age, who also happen to be the sickest and most likely to require complicated care. This is further exacerbated by the fact that 10% of Americans aged >65 have diagnosed dementia, and 1 in 3 patients aged >65 have some degree of cognitive impairment, with likely negative impact on their computer literacy.^27^

With what we can call problematic and increasingly complex EMR and medical data management systems challenging patients and healthcare workers alike, the time patients are able to spend with their health care providers is continuously being chipped away. This makes the future for implementing artificial intelligence (AI) likely to be adopted as a new normal by younger physicians who, during their training, likely had little exposure to the Hippocratic patient-physician relationship, instead being immersed into a new ‘computer-patient’ relationship, with disappearance of human driven physician-to-patient connections. With this reality already upon us, we must ask if there is any room left for the *art of oncology* in this brave new world, focused on ‘state-of-the art’ computer-assisted, and sooner rather than later, AI-driven clinical medicine. Will we retain the doctor as healer, artist and magician as we like to be viewed by the patient? Or will we now transition to the physician becoming a pilot and computer wizard or, in other words, a drone pilot, where the drone is the patient, and we as physicians will have as much interaction with the patients as the military drone pilots have with their drones operated across the globe, sometimes never setting eyes on the actual physicality of the machine, with everything being done via the computer?.

We acknowledge the risk of us showing our age and closeness to retirement when maintaining our belief that the *art of oncology* cannot be practiced by a robot, no matter how sophisticated the AI becomes. If future physicians lose the skills outlined in table 1, they become no different from a commercial airline pilot who, with great skills, safely gets passengers from point A to point B, but never sees or interacts with their passengers. But patients who are sick and need healing are not airline passengers or drones, they are our fellow human beings with events that negatively impact their quality of life. Even though computer-aided diagnoses and treatment algorithms are rightfully viewed as a blessing by most, it should not be lost that the empathy and wisdom that only an *artist of medicine* can master, can turn even the most serious medical situation into healing and comfort. We must recognize that, if we continue to yield to our new ‘superior’ ‘*Dr. AI and its team of computers*’, *the art of oncology* will certainly be lost!

In conclusion, we believe that if we want to try and retain some part of the *art of oncology* and medicine in general, we need to strive to achieve the skillset outlined in table 1 and, in that process, try to reverse the current trend of reducing FTF time between patients and physicians. Additionally, below are some suggestions to what we, as individual physicians, can do to protect our FTF involvement with our patients:

- Become more critical and challenge our non-clinical administrators, politicians and computer engineers when new mandates and regulations that invade the patient-provider relationship are issued through laws and ‘software updates’.
- Have meetings with patients without being distracted by a computer, tablet or cell phone.
- Think, and then talk with colleagues, patients, and administrators about the impact that unrelenting computerisation has on patients’ wellbeing and, if necessary, recite in our mind our Hippocratic oath on how we should conduct ourselves in our relationship with patients.
